# Comparative evaluation of three immunochromatographic identification tests for culture confirmation of *Mycobacterium tuberculosis* complex

**DOI:** 10.1186/1471-2334-14-54

**Published:** 2014-02-01

**Authors:** Kinuyo Chikamatsu, Akio Aono, Hiroyuki Yamada, Tetsuhiro Sugamoto, Tomoko Kato, Yuko Kazumi, Kiyoko Tamai, Hideji Yanagisawa, Satoshi Mitarai

**Affiliations:** 1 Department of Mycobacterium Reference and Research, Research Institute of Tuberculosis, Japan Anti-Tuberculosis Association, Kiyose, Tokyo 204-8533, Japan; 2 Department of Basic Mycobacteriosis, Nagasaki University Graduate School of Biomedical Sciences, Nagasaki 852-8501, Japan; 3 Miroku Medical Laboratory Company Limited, 659-2 Innai, Saku, Nagano 384-2201, Japan

**Keywords:** Capilia TB-Neo, *Mycobacterium tuberculosis* complex identification, *mpt64* gene

## Abstract

**Background:**

The rapid identification of acid-fast bacilli recovered from patient specimens as *Mycobacterium tuberculosis* complex (MTC) is critically important for accurate diagnosis and treatment. A thin-layer immunochromatographic (TLC) assay using anti-MPB64 or anti-MPT64 monoclonal antibodies was developed to discriminate between MTC and non-tuberculosis mycobacteria (NTM). Capilia TB-Neo, which is the improved version of Capilia TB, is recently developed and needs to be evaluated.

**Methods:**

Capilia TB-Neo was evaluated by using reference strains including 96 *Mycobacterium* species (4 MTC and 92 NTM) and 3 other bacterial genera, and clinical isolates (500 MTC and 90 NTM isolates). *M. tuberculosis* isolates tested negative by Capilia TB-Neo were sequenced for *mpt64* gene.

**Results:**

Capilia TB-Neo showed 100% agreement to a subset of reference strains. Non-specific reaction to *M. marinum* was not observed. The sensitivity and specificity of Capilia TB-Neo to the clinical isolates were 99.4% (99.6% for *M. tuberculosis,* excluding *M. bovis* BCG) for clinical MTC isolates and 100% for NTM isolates tested, respectively. Two *M. tuberculosis* isolates tested negative by Capilia TB-Neo: one harbored a 63-bp deletion in the *mpt64* gene and the other possessed a 3,659-bp deletion from Rv1977 to Rv1981c, a region including the entire *mpt64* gene.

**Conclusions:**

Capilia TB-Neo is a simple, rapid and highly sensitive test for identifying MTC, and showed better specificity than Capilia TB. However, Capilia TB-Neo still showed false-negative results with *mpt64* mutations. The limitation should be recognized for clinical use.

## Background

Tuberculosis remains a major threat to global health, and therefore, rapid identification of the causative *M. tuberculosis* complex (MTC) is critical. Liquid culture detection is now widely used for managing HIV-co-infected and drug-resistant tuberculosis, and liquid culture can improve the recovery of acid-fast bacilli and decreases the time to detection. However, because other non-tuberculosis mycobacterium (NTM) species may also grow, it is important to identify MTC from positive culture for rapid and appropriate management of tuberculosis.

Several methods are available to identify mycobacteria. Conventional biochemical tests are generally time-consuming [1,2] and not surely reproducible, while more recently developed techniques involving molecular biology [3-7] or high-performance liquid chromatographic analysis of mycolic acid [8] are accurate and rapid, but these require expensive devices. In contrast, immunochromatographic species identification tests, Capilia TB (TAUNS, Izunokuni, Japan), SD BIOLINE TB Ag MPT64 rapid (Standard Diagnostics, Inc., Korea) and BD MGIT™ TBc Identification Test (Becton, Dickinson and Company, USA) have been adopted as a cheap, rapid, and accurate alternative in clinical laboratories around the world [9-11]. However, false positives to *Mycobacterium marinum*, *Staphylococcus aureus*[9,12], and false negatives in MPB64 mutants [10,13] have occasionally been reported. Capilia TB-Neo (TAUNS, Izunokuni, Japan), an improved version of Capilia TB, has recently been developed to overcome these problems. In this study, we evaluated the performance of Capilia TB-Neo with reference strains and clinical isolates. Any false-negative MTC clinical isolate detected by Capilia TB-Neo were further investigated relative genes.

## Methods

### Reference strains and clinical isolates

Reference strains of 96 *Mycobacterium* species and subspecies (4 MTC and 92 NTM) and 3 other genera with acid-fastness (*Nocardia asteroids*, *Rhodococcus equi* and *Rhodococcus aichiense*) were used for the evaluation (Table 1). A total of 500 MTC and 90 NTM clinical isolates (10 *M. abscessus*, 4 *M. chelonae*, 13 *M. fortuitum*, 8 *M. gordonae*, 15 *M. avium* complex, 7 *M. intracellulare*, 3 *M. nonchromogenicum*, 5 *M. scrofulaceum*, 4 *M. xenopi*, 15 *M. kansasii*, 1 *M. gastri*, 2 *M. peregrinum*, 1 *M. intermedium*, 1 *M. szulgai*, and 1 *M. marinum*) were selected to provide a representative sample of the isolates available from Miroku Medical Laboratory Co., Ltd. (Saku, Japan) from 2009 to 2010, and the collection from the Ryoken survey in 2002 and 2007. The clinical isolates were collected from patients as a part of routine examination. No ethical approval was required for this type of laboratory based study only using isolates. Reference strains and clinical isolates were cultured with OADC-supplemented Middlebrook 7H9 broth (Becton, Dickinson and Company, USA) and 2% Ogawa medium (Kyokuto Pharmaceutical Industrial Co., Japan) at 37°C or 30°C.

**Table 1:** List of reference strains and the results of identification of MTC by using Capilia TB-Neo, SD MPT64, and TBc ID

**Species**	**Strain**	**Capilia TB-Neo**	**SD MPT64**	**TBc ID**	**Species**	**Strain**	**Capilia TB-Neo**	**SD MPT64**	**TBc ID**
*M. tuberculosis* H37Rv	ATCC27294	+	+	+	*M. interjectum*	ATCC51457	-	-	-
*M. africanum*	ATCC25420	+	+	+	*M. intermedium*	ATCC51848	-	-	-
*M. bovis*	ATCC19210	+	+	+	*M. intracellulare*	ATCC13950	-	-	-
*M. microti*	ATCC19422	+	+	+	*M. kansasii*	ATCC12478	-	-	-
*M. abscessus*	ATCC19977	-	-	-	*M. kubicae*	ATCC700732	-	-	-
*M. acapulcensis*	ATCC14473	-	-	-	*M. lactis*	ATCC27356	-	-	-
*M. agri*	ATCC27406	-	-	-	*M. lentiflavum*	ATCC51985	-	-	-
*M. aichiense*	ATCC27280	-	-	+	*M. madagascariense*	ATCC49865	-	-	-
*M. alvei*	ATCC51304	-	-	-	*M. malmoense*	ATCC29571	-	-	-
*M. asiaticum*	ATCC25276	-	-	-	*M. marinum*	ATCC00927	-	-	+
*M. aurum*	ATCC23366	-	-	-	*M. moriokaense*	ATCC43059	-	-	-
*M. austroafricanum*	ATCC33464	-	-	-	*M. mucogenicum*	ATCC49650	-	-	-
*M. avium* subsp. *avium*	ATCC25291	-	-	-	*M. neoaurum*	ATCC25795	-	-	-
*M. avium* subsp. *paratuberculosis*	ATCC19698	-	-	-	*M. nonchromogenicum*	ATCC19530	-	-	-
*M. avium* subsp. “*suis”*	ATCC19978	-	-	-	*M. novum*	ATCC19619	-	-	-
*M. avium* subsp. *silvaticum*	ATCC49884	-	-	-	*M. obuense*	ATCC27023	-	-	-
*M. branderi*	ATCC51789	-	-	-	*M. paraffinicum*	ATCC12670	-	-	-
*M. brumae*	ATCC51384	-	+	-	*M. parafortuitum*	ATCC19686	-	-	-
*M. celatum*	ATCC51131	-	-	-	*M. peregrinum*	ATCC14467	-	-	-
*M. celatum II*	ATCC51130	-	-	-	*M. petroleophilum*	ATCC21497	-	-	-
*M. chelonae chemovar niacinogenes*	ATCC35750	-	-	-	*M. phlei*	ATCC11758	-	-	-
*M. chelonae* subsp. *chelonae*	ATCC35752	-	-	-	*M. porcinum*	ATCC33776	-	-	-
*M. chitae*	ATCC19627	-	-	+	*M. poriferae*	ATCC35087	-	-	-
*M. chlorophenolicum*	ATCC49826	-	-	-	*M. pulveris*	ATCC35154	-	-	-
*M. chubuense*	ATCC27278	-	-	-	*M. rhodesiae*	ATCC27024	-	-	-
*M. confluentis*	ATCC49920	-	-	-	*M. scrofulaceum*	ATCC19981	-	-	-
*M. conspicuum*	ATCC700090	-	-	-	*M. senegalense*	ATCC35796	-	-	-
*M. cookii*	ATCC49103	-	-	-	*M. septicum*	ATCC700731	-	-	-
*M. diernhoferi*	ATCC19340	-	-	-	*M. shimoidei*	ATCC27962	-	-	-
*M. duvalii*	ATCC43910	-	-	-	*M. shinshuense*	ATCC33728	-	-	-
*M. engbaekii*	ATCC27353	-	-	-	*M. simiae*	ATCC25275	-	-	-
*M. flavescens*	ATCC14474	-	-	-	*M. smegmatis*	ATCC19420	-	-	-
*M. fortuitum* subsp. *acetamidolyticum*	ATCC35931	-	-	-	*M. smegmatis*	ATCC700084	-	-	-
*M. fortuitum* subsp. *fortuitum*	ATCC06841	-	-	-	*M. sphagni*	ATCC33027	-	-	-
*M. fortuitum* subsp. *fortuitum*	ATCC49403	-	-	-	*M. szulgai*	ATCC35799	-	-	-
*M. gadium*	ATCC27726	-	-	+	*M. terrae*	ATCC15755	-	-	-
*M. gallinarum*	ATCC19710	-	-	-	*M. terrae*	DSMZ43540	-	-	-
*M. genavense*	ATCC51234	-	-	-	*M. terrae*	DSMZ43541	-	-	-
*M. gilvum*	ATCC43909	-	-	-	*M. terrae*	DSMZ43542	-	-	-
*M. goodii*	ATCC700504	-	-	-	*M. thermoresistibile*	ATCC19527	-	-	-
*M. gordonae*	ATCC14470	-	-	-	*M. tokaiense*	ATCC27282	-	-	-
*M. gordonae group B*^*19*^	KK33-08	-	-	-	*M. triplex*	ATCC700071	-	-	-
*M. gordonae group C*^*19*^	KK33-53	-	-	-	*M. triviale*	ATCC23292	-	-	-
*M. gordonae group D*^*19*^	KK33-46	-	-	-	*M. vaccae*	ATCC15483	-	-	-
*M. haemophilum*	ATCC29548	-	-	-	*M. valentiae*	ATCC29356	-	-	-
*M. hassiacum*	ATCC700660	-	-	-	*M. wolinskyi*	ATCC700010	-	-	-
*M. heckeshornense*	DSMZ44428	-	-	-	*M. xenopi*	ATCC19250	-	-	-
*M. heidelbergense*	ATCC51253	-	-	-	*Nocardia asteroides*	ATCC19247	-	-	-
*M. hiberniae*	ATCC49874	-	-	-	*Rhodococcus equi*	ATCC6939	-	-	-
					*Rhodococcus aichiense*	ATCC33611	-	-	-

### Identification of mycobacteria

*Mycobacterium* species of the clinical isolates were identified using one or more of the following approaches: (i) the DNA or RNA amplification kits Cobas Amplicor PCR (Roche Diagnostics, Japan) and TRC Rapid (Tosoh Bioscience, Japan); (ii) the DNA-DNA hybridization DDH Mycobacteria Kit (Kyokuto Pharmaceutical Industrial Co., Japan); and (iii) 16S rRNA gene sequencing, supplementary [7]. The isolates identified as MTC were further examined by multiplex PCR analysis of *cfp32*, the region of difference (RD) 9, and RD12 according to the method of Nakajima et al. [14]. When MTC species other than *M. tuberculosis* sensu stricto were detected, they were further characterized with respect to RD1, RD4, RD7, and MiD3 [15]. If *M. bovis* Bacillus Calmette-Guerin (BCG) was identified, additional multiplex PCR analyses were performed to test for RD2, RD14, RD15, RD16, and *SenX3-RegX3* to distinguish sub-strains of BCG [16,17]. The multiplex PCR amplification was performed using a Type-it Microsatellite PCR Kit (QIAGEN, Japan). Each PCR reaction contained 1.0 μl of DNA template, 6.25 μl of Type-it multiplex PCR Master mix, 1.25 μl of Q-solution, 0.25 μl of each primer (10 pmol/μl) and an appropriate amount of molecular grade water for a total reaction volume of 13 μl. The thermal profile was as follows: (i) 95°C (5 min); (ii) 28 cycles of 95°C (0.5 min), 58 or 55°C (1.5 min), 72°C (0.5 min); and (iii) a final extension step at 68 or 60°C (10 or 30 min). The amplified products were analyzed by 3% agarose gel electrophoresis. The expected RD loci for each MTC species are summarized in Table 2.

**Table 2:** Oligonucleotide primers used in PCR and direct sequencing

**Target gene**	**Primer ID**	**Nucleotide sequence (5'-3')**	**Size (bp)**	**Ref. no.**
MTC identification				
16S rRNA	285	GAGAGTTTGATCCTGGCTCAG	1028	7
	264	TGCACACAGGCCACAAGGGA		
	259	TTTCACGAACAACG GACAA	591	
*cfp32*	Rv0577F	ATGCCCAAGAGAAGCGAATACAGGCAA	786	14
	Rv0577R	CTATTGCTGCGGTGCGGGCTTCAA		
RD9	Rv2073cF	TCGCCGCTGCCAGATGAGTC	600	14
	Rv2073cR	TTTGGGAGCCGCCGGTGGTGATGA		
RD12	Rv3120F	GTCGGCGATAGACCATGAGTCCGTCTCCAT	404	14
	Rv3120R	GCGAAAAGTGGGCGGATGCCAG		
RD1	ET1	AAGCGGTTGCCGCCGACCGACC		15
	ET2	CTGGCTATATTCCTGGGCCCGG		
	ET3	GAGGCGATCTGGCGGTTTGGGG		
RD4	Rv1510F	GTGCGCTCCACCCAAATAGTTGC	1033	15
	Rv1510R	TGTCGACCTGGGGCACAAATCAGTC		
RD7	Rv1970F	GCGCAGCTGCCGGATGTCAAC	1116	15
	Rv1970R	CGCCGGCAGCCTCACGAAATG		
MiD3	IS1561F	GCTGGGTGGGCCCTGGAATACGTGAACTCT	530	15
	IS1561R	AACTGCTCACCCTGGCCACCACCATGGACT		
Distinguish sub-strains of BCG				
RD2	RD2l	CCAGATTCAAATGTCCGACC		16
	RD2r	GTGTCATAGGTGATTGGCTT		
RD14	RD14l	CAGGGTTGAAGGAATGCGTGTC		16
	RD14r	CTGGTACACCTGGGGAATCTGG		
RD15	RD8l	ACTCCTAGCTTTGCTGTGCGCT		16
	RD8r	GTACTGCGGGATTTGCAGGTTC		
RD16	RD16nf	ACATTGGGAAATCGCTGCTGTTG		17
	RD16nr	GGCTGGTGTTTCGTCACTTC		
*SenX3-RegX3*	C3	GCGCGAGAGCCCGAACTGC		16
	C5	GCGCAGCAGAAACGTCAGC		
Sequencing				
*mpt64* (Rv1980c)	mpb64W-F	ACTCAGATATCGCGGCAATC	1061	this study
	mpb64W-R	CGATCACCTCACCTGGAGTT		
Rv1977	Rv1977F	GTTTCCCGAGATCAGCTCAA	348	this study
	Rv1977R	ATCTCGTCGTGTGTCACCAG		
Rv1981c	Rv1981F	GATCGAATGCAGGCTGGTAT	399	this study
	Rv1981R	ACTACTACCGCGGTGACGAC		

### Capilia TB-Neo, SD MPT64, and TBc ID

The validation of Capilia TB-Neo (TAUNS, Izunokuni, Japan) was conducted using the aforementioned reference strains as well as MTC and NTM clinical isolates. In addition, SD BIOLINE TB Ag MPT64 rapid (SD MPT64: Standard Diagnostics, Inc. Korea) and BD MGIT™ TBc Identification Test (TBc ID: Becton, Dickinson and Company, USA), detect MPT64 which is the same as MPB64, were tested using reference strains and NTM clinical isolates. Each test was performed according to the manufacturer’s instructions. Briefly, clinical isolates growing on Ogawa medium were suspended in 1 ml of sterile distilled water, and the suspension subjected to the test. Similarly, positive liquid cultures of reference strains (McFarland No. 1 to 2) were directly subjected to each test. Positive test results were indicated by a red line in the test area after 15 min.

### Sequencing of the *mpt64* gene

Any false-negative *M. tuberculosis* isolate detected by Capilia TB-Neo was further analyzed by sequencing *mpt64* and surrounding genes by using the primers listed in Table 2. Each PCR reaction contained 1.0 μl of DNA template, 12.5 μl of Type-it multiplex PCR Master mix, 2.5 μl of Q-solution, 0.5 μl of each primer (10 pmol/μl) and an appropriate amount of molecular grade water for a total reaction volume of 25 μl. The thermal profile was as follows: (i) 95°C (5 min); (ii) 30 cycles of 95°C (0.5 min), 62°C (1.5 min), 72°C (1 min); and (iii) final extension at 60°C (10 min). The amplified product was analyzed by 3% agarose gel electrophoresis and was purified using Mag Extractor (TOYOBO, Japan). The purified DNA products were subjected to direct sequencing using an ABI 377 automatic sequencer (Applied Biosystems, USA) and BigDye Terminator Cycle Sequencing v 3.1 (Applied Biosystems, USA), according to the manufacturer’s instructions. DNA sequences of *mpt64* from each isolate were compared with *M. tuberculosis* H37Rv by using Genetyx-win ver. 5.2 (Genetyx Co., Japan).

## Results

Each of the three kits (Capilia TB-Neo, SD MPT64, and TBc ID) was tested using the 99 reference strains. Capilia TB-Neo correctly produced positive results for four MTC (*M. tuberculosis*, *M. africanum*, *M. bovis,* and *M. microti*) and negative results for 92 NTM and 3 non-mycobacterial species (other genera) with acid-fastness, while SD MPT64 and TBc ID generated several false positives (Table 1). The sensitivity and specificity of Capilia TB-Neo to reference strains were 100%.

Of the 500 MTC clinical isolates tested, 497 were identified as MTC by Capilia TB-Neo. The other 3 isolates that tested negative by using Capilia TB-Neo also tested negative by using SD MPT64 and TBc ID. All three kits produced negative results for all 90 NTM clinical isolates examined. Thus, The sensitivity and specificity of Capilia TB-Neo to the clinical isolates were 99.4% and 100%, respectively.

**Figure 1:**
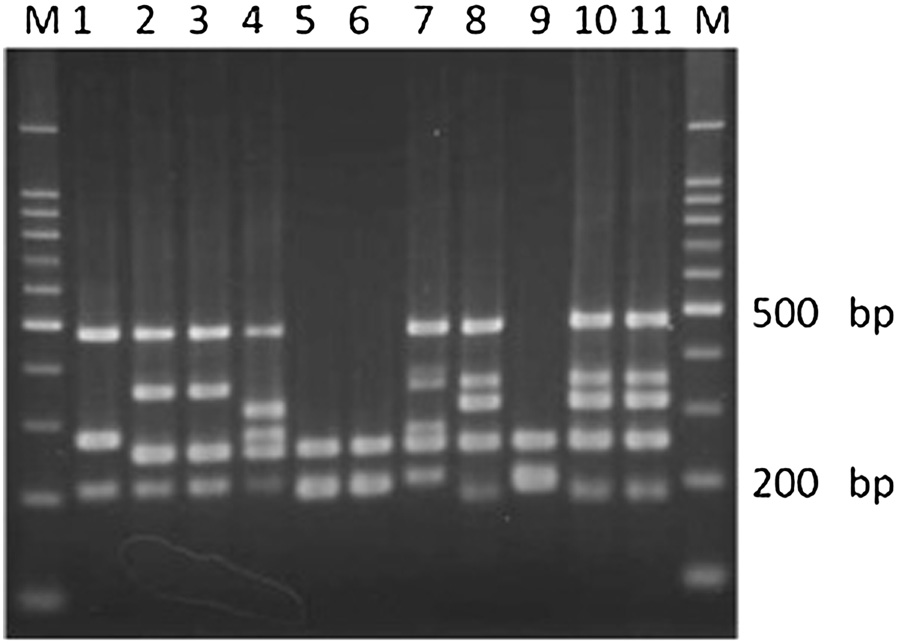
**Multiplex PCR analysis of *Mycobacterium bovis *BCG sub-strains and clinical isolates.** 1: BCG Pasteur (ATCC35734), 2: BCG Glaxo (ATCC35741), 3: BCG Copenhagen (ATCC27290), 4: BCG Russian (ATCC35740), 5: BCG Montreal (ATCC35735), 6: BCG Connaught (ATCC35745), 7: BCG Danish, 8: BCG Tokyo, 9: Sample 421, 10: Sample 467, 11: Sample 475, M: Size marker.

The multiplex PCR system identified 492 *M. tuberculosis* isolates out of 500. Five isolates, which were *cfp32*-, RD9-, RD4-, RD7-, and MiD3-positive, but RD12*-*negative, were initially identified as *M. canettii*. However, colonies of these isolates showed a consistent rough surface on solid medium, and subsequent sequencing of *hsp65* indicated that the isolates had the genotype of *M. tuberculosis* sensu stricto (data not shown). These isolates were collected from different areas of Japan. Consequently, 497 isolates were identified as *M. tuberculosis*. The remaining 3 isolates were deficient in RD1, RD4, RD7, RD9, and RD12, and therefore were identified as *M. bovis* BCG. Two of these isolates were confirmed as *M. bovis* BCG Tokyo based on the unique size of RD16, and the third isolate had the same RD pattern as BCG Connaught and BCG Montreal, as for RD2, RD14, RD15, RD16 and *SenX3-RegX3* (Figure 1). Among the 3 MTC isolates that tested negative by Capilia TB-Neo, 2 isolates were *M. tuberculosis* and the other was *M. bovis* BCG Connaught or BCG Montreal (Table 3).

**Table 3:** Results of PCR detection and Capilia TB-Neo of MTC with clinical isolates

**Species interpretation (Number of isolates)**	**Banding pattern**							**Capilia TB-Neo**	**%**
	***cfp32***	**RD9**	**RD12**	**RD4**	**RD7**	**MiD3**	**RD1**		
*M. tuberculosis* (490)	+	+	+	NT	NT	NT	NT	+	98.0
*M. tuberculosis* (2)	+	+	+	NT	NT	NT	NT	-	0.4
*"M. canettii"* (5)^a^	+	+	-	+	+	+	+	+	1.0
*M. bovis* BCG Tokyo (2)^b^	+	-	-	-	-	+	-	+	0.4
*M. bovis* BCG Connaught (1)^c^	+	-	-	-	-	+	-	-	0.2

^a^Confirmed to be *M. tuberculosis* by *hsp65*  sequencing and morphology, ^b^Confirmed by contracted RD16, ^c^Confirmed by absence of RD2 and RD15, and contracted *SenX3-RegX3*, NT: Not tested.

Mutations in the *mpt64* gene were detected by sequencing two *M. tuberculosis* isolates with negative results by Capilia TB-Neo. One isolate had a deletion of 63 bp from nucleotides 196 to 258 (amino acids position 43 to 63), and the other had a deletion of 3,659 bp from nucleotide 874 in Rv1977 to nucleotide 905 in Rv1981c, which included the whole *mpt64* gene.

## Discussion

In many industrialized countries, the ability to rapidly distinguish between MTC and NTM is critical in clinical practice. Indeed, the anti-tuberculosis drug resistance survey in Japan revealed that 19.3% of all clinical mycobacterial isolates are NTM [18], underscoring the importance of rapid and accurate detection of MTC from acid-fast bacillus-positive culture. The immunochromatographic assay kit for the identification of MTC is now widely used in many countries. Capilia TB-Neo is the improved version of Capilia TB, and has been subjected to few clinical evaluations. Here, we report good overall performance of the kit but with several limitations.

In this study, the sensitivity of Capilia TB-Neo was 99.4% to clinical MTC isolates or 99.6% excluding *M. bovis* BCG, while the specificity of the kit tested to clinical NTM isolates was 100%. However, the isolation of BCG could present a practical problem. The *M. bovis* BCG Tokyo strain is sporadically isolated in Japan as a complex of vaccination or bladder cancer therapy, and is identified as MTC with the kit [19]. Some BCG strains such as Connaught, Pasteur, and Tice lack RD2 including the *mpt64* gene, but RD2 is conserved in others such as Tokyo, Moreau, and Russia [16]. This issue should be properly addressed to avoid confusion. Although it is difficult to discriminate BCG Tokyo from MTC with *mpt64*/*mpb64*, their differentiation would be an important advance in the development of a future TLC product. The weak false-positive reaction to *M. marinum* that was reported using Capilia TB [12] was not observed in this study, and resulted in better specificity. The minimum detection concentration of *M. tuberculosis* for Capilia TB-Neo was 10^5^ CFU/ml (data not shown), which was one-tenth than that for the previous kit. There was a report that Cpilia TB-Neo was higher sensitivity than Capilia TB [20]. In summary, the overall performance of Capilia TB-Neo was better than Capilia TB in both sensitivity and specificity.

SD MPT64 and TBc ID were also tested with reference strains. Both SD MPT64 and TBc ID showed false-positive results against several NTM strains in this study. Kodama et al. [12] reported that no *M. marinum* strains grown on 2% Ogawa medium tested positive by using the Capilia TB, while all strains grown on 3 kinds of liquid medium, MGIT (Becton Dickinson, Japan), KRD medium (Japan BCG Laboratory, Japan) and Myco Acid (Kyokuto Pharmaceutical Industrial Co. Ltd., Japan), eventually displayed a positive reaction that intensified with time. Kodama et al. speculated that nonspecific antigen which could make complex with anti-MPB64 antibody may be produced in liquid mediums, but not on solid medium. Considering the effect of liquid culture, the original bacterial suspensions giving false-positive results, that were prepared from liquid and solid culture, were then re-tested before and after 10-fold dilution. Interestingly, none of these diluted strains tested positive in these kits, but bacterial concentrations were high enough for positive results in case of MTC. These results implied that a high concentration of bacterial antigens could induce non-specific reactions in SD MPT64 and TBc ID. The manufacturer’s instructions for the TBc ID indicate that this kit may be used up to 10 days after a positive MGIT alarm. This non-specific reaction should be properly addressed in clinical practice, and the users should perform morphological characterization with a microscope to identify cord formation.

Several mutations in the *mpt64* gene produce a negative test result for *M. tuberculosis* isolates in the TLC assay using anti-MPB64 monoclonal antibodies. To date, these include a 63-bp deletion from nucleotide 196, a 1-bp deletion from nucleotide 266, a point mutation at position 388 or 402, *IS6110* insertion mutation at position 177 or 501, a 176-bp deletion from nucleotide 512, and a 1-bp insertion at position 287 [10,13,21]. In our study, 2 *M. tuberculosis* isolates gave false-negative results by using the Capilia TB-Neo, SD MPT64, and TBc ID. One isolate had a deletion of 63 bp from nucleotide 196 in the *mpt64* gene as reported previously, and the other isolate possessed a 3,659-bp deletion from nucleotide 874 in Rv1977 to 905 in Rv1981c, including the whole *mpt64* gene. To the best of our knowledge, this is the first report of a large deletion in *mpt64*. A transposon site hybridization (TraSH) study [22] indicated that *mpt64* is not essential for infection or *in vitro* growth of *M. tuberculosis*. This large deletion mutant supported the finding.

In summary, the TLC assay detecting MPB64 or MPT64 can be applied to specimens prepared from liquid and solid culture. It does not need special reagents, instruments, or complex techniques. Capilia TB-Neo tested in this study showed excellent sensitivity with perfect specificity.

## Conclusions

Capilia TB-Neo showed high sensitivity and specificity with clinical mycobacterial isolates, and 100% specificity to reference strains. However, 2 *M. tuberculosis* isolates were tested negative by Capilia TB-Neo because of mutations in the *mpt64* gene, and positive to certain BCG sub-strain. This study, therefore, serves to emphasize the importance of careful use of the kit and the complementary techniques such as morphological identification.

## Competing interests

The authors declare that they have no competing interests.

## Authors’ contributions

KC carried out the TLC assays, molecular genetic studies, sequence alignment and drafted the manuscript. AA, HY, TS, and TK cultured clinical isolates. HY helped to draft the manuscript. YK prepared reference strains. KT and HY collected clinical isolates. SM was responsible for planning the study. All authors read and approved the final manuscript.

## Pre-publication history

The pre-publication history for this paper can be accessed here:

http://www.biomedcentral.com/1471-2334/14/54/prepub
